# Supplementary material for: Subcellular compartmentalization of the plant antioxidant system: an integrated overview
Source: PeerJ. 2020 Jul 16;8:e9451. doi: 10.7717/peerj.9451 (PMC7369019; doi:10.7717/peerj.9451)
Supplement: Supplemental Information 1 [file peerj-08-9451-s001.docx]

Table S1 Specific search terms for the review sections and number of total and selected articles.

| **Review section name** | **Specific search terms** | **Total number of articles** | **Number of selected articles** |
| --- | --- | --- | --- |
| Main features of the antioxidant system components | catalase, glutathione peroxidase, ascorbate peroxidase, superoxide dismutase, ascorbate-glutathione cycle, monodehydroascorbate reductase, dehydroascorbate reductase, glutathione reductase, ascorbate, glutathione, glutathione disulfide, reaction mechanism, enzyme kinetic | 584 | 29 |
| Experimental data concerning the antioxidant system components | experimental measurement, parameters, quantification, concentration, reaction rate, dynamics, biochemical methods, stress response, ascorbate content, glutathione content, stress tolerance, drought stress, salt stress, cold stress, abiotic, biotic, regulation | 2910 | 25 |
| Stress-response of the antioxidant system components | signaling pathway, hormones, regulators, inductors, activity, abiotic stress, biotic stress, regulation, stress combination effect | 165 | 30 |
| Subcellular compartmentalization of the antioxidant system components | compartmentalization, chloroplastic, mitochondrial, peroxisomal, subcellular distribution, chloroplast redox status, electron transport chain, vacuole | 217 | 32 |
| Modeling approaches to study the antioxidant system | ascorbate-glutathione cycle models, chloroplastic antioxidant models, mathematical models, computational | 74 | 5 |

* Common search terms: antioxidant system, reactive oxygen species, plant
